# Diagnostic accuracy of noninvasive optical imaging for basal cell carcinoma: A systematic review and meta-analysis

**DOI:** 10.1016/j.jdin.2026.06.026

**Published:** 2026-08-01

**Authors:** Bella A. Kelemen, Koorosh Karimi, Lili Gulyás, Fanni A. Meznerics, Basak Danisan, Karen K. Fazekas, Péter Hegyi, Kende K. Lőrincz, András Bánvölgyi, Norbert Wikonkál, Norbert Kiss

**Affiliations:** a Department of Dermatology, Venereology and Dermatooncology, Faculty of Medicine, Semmelweis University, Budapest, Hungary; b Centre for Translational Medicine, Semmelweis University, Budapest, Hungary; c Institute for Translational Medicine, Medical School, University of Pécs, Pécs, Hungary; d Institute of Pancreatic Diseases, Semmelweis University, Budapest, Hungary; e Central Hospital of Northern Pest-Military Hospital, Budapest, Hungary

**Keywords:** basal cell carcinoma, diagnostic accuracy, line-field confocal optical coherence tomography, noninvasive imaging, optical coherence tomography, reflectance confocal microscopy

*To the Editor:* Basal cell carcinoma (BCC) is the most common type of cancer.^1^ Although it rarely metastasizes, delayed diagnosis and treatment may cause significant local tissue destruction.^2^ Histopathologic examination remains the diagnostic gold standard for BCC; however, it is invasive and may delay treatment.^3^ Dermoscopy is widely used as a first-line non-invasive diagnostic tool in dermatological practice, while additional imaging techniques may support the diagnostic process in clinically or dermoscopically equivocal lesions.^3^ Several real-time noninvasive optical imaging techniques have been introduced, including optical coherence tomography (OCT), reflectance confocal microscopy (RCM), and line-field optical coherence tomography (LC-OCT).^4^ Given their capacity for detailed morphologic assessment, this study focuses specifically on evaluating the diagnostic accuracy of these techniques for confirming BCC in equivocal lesions.

A systematic review and meta-analysis were conducted following the Cochrane Handbook recommendations and the PRISMA 2020 statement.^5^ The protocol was registered in PROSPERO (CRD42024604297). Searches were performed in major databases through October 2024. Studies evaluating the diagnostic accuracy of non-invasive optical imaging techniques in patients with suspected BCC using histopathology as the reference standard were included.

Twenty-nine studies were eligible, of which 26 were included in the quantitative analysis. Pooled sensitivity and specificity were estimated using a bivariate binomial generalized linear mixed model.

LC-OCT demonstrated the highest pooled sensitivity (0.97; 95% CI, 0.95-0.98) with high specificity (0.93; 95% CI, 0.85-0.97). RCM also showed high accuracy, with pooled sensitivity of 0.95 (95% CI, 0.88-0.98, I^2^ = 85%) and specificity of 0.95 (95% CI, 0.90-0.98, I^2^ = 79%). OCT demonstrated high sensitivity (0.92; 95% CI, 0.88-0.94, I^2^ = 57%), although specificity was lower (0.84; 95% CI, 0.77-0.89, I^2^ = 72%). Corresponding predictive values are summarized in Table I.

**Table I:** Pooled sensitivity, specificity, PPV, NPV, and diagnostic odds ratio of detecting BCC by LC-OCT, RCM and OCT

Diagnostic tool	Sensitivity	Specificity	PPV∗	NPV∗	DOR	Level of evidence for sensitivity	Level of evidence for specificity
LC-OCT	0.97 (0.95-0.98)	0.93 (0.85-0.97)	0.977	0.919	494.3 (208.9-1169.9)	⨁⨁⨁◯ Moderate	⨁⨁⨁◯ Moderate
RCM	0.95 (0.88-0.98)	0.95 (0.90-0.98)	0.924	0.967	403.2 (146.3-1111.4)	⨁⨁◯◯ Low	⨁⨁◯◯ Low
OCT	0.92 (0.88-0.94)	0.84 (0.77-0.89)	0.863	0.904	57.4 (39.1-84.1)	⨁⨁⨁⨁ High	⨁⨁⨁◯ Moderate

*DOR*, Diagnostic odds ratio; *LC-OCT*, line-field optical coherence tomography; *NPV*, negative predictive value; *OCT*, optical coherence tomography; *PPV*, positive predictive value; *RCM*, reflectance confocal microscopy.

∗ Calculated based on the pooled prevalence of the included articles.

Overall, LC-OCT and RCM demonstrated excellent diagnostic performance, with sensitivity and specificity values above 90%, supporting their role as second-step diagnostic tools in equivocal lesions, providing near-histological resolution to further characterize lesions. Importantly, rather than replacing biopsy, these techniques should be understood as tools that refine lesion stratification after dermoscopic assessment. They may help identify cases that do not require immediate histopathologic sampling, while biopsy remains necessary for persistently equivocal lesions. In contrast, OCT showed lower specificity and lower predictive values, particularly positive predictive value, limiting its reliability for definitive lesion confirmation, but it may still provide information regarding lesion depth and tumor architecture in selected cases. Rather than representing competing methods, these techniques may provide complementary information within the diagnostic pathway of BCC. Their integration into clinical practice may improve diagnostic triage, reduce unnecessary biopsies, and support timely decision-making.

Some limitations should be acknowledged. The number of eligible studies was limited for LC-OCT, and moderate heterogeneity was observed. Differences in study design and disease prevalence may also influence pooled estimates. Furthermore, the analysis was limited to diagnostic confirmation; subtype differentiation and treatment monitoring were beyond the scope of this study.

In conclusion, LC-OCT and RCM demonstrate high diagnostic accuracy for the detection of basal cell carcinoma and may support noninvasive diagnostic decision-making in dermatological practice.

### Declaration of generative AI and AI-assisted technologies in the writing process

During the preparation of this manuscript, the author(s) used ChatGPT (OpenAI, GPT-5) to assist with language editing and improving the clarity of the text. The author(s) carefully reviewed and edited the generated content as necessary and take full responsibility for the final content of the manuscript.

## Conflicts of interest

None disclosed.
